# The independence of deficits in position sense and visually guided reaching following stroke

**DOI:** 10.1186/1743-0003-9-72

**Published:** 2012-10-04

**Authors:** Sean P Dukelow, Troy M Herter, Stephen D Bagg, Stephen H Scott

**Affiliations:** 1 Department of Clinical Neurosciences, Hotchkiss Brain Institute, University of Calgary, Calgary, AB, Canada; 2 Centre for Neuroscience Studies, Queen’s University, Kingston, ON, Canada; 3 Department of Exercise Science, University of South Carolina, Columbia, SC, USA; 4 Providence Care, St. Mary’s of the Lake Hospital, Kingston, ON, Canada; 5 Department of Physical Medicine and Rehabilitation, Queen’s University, Kingston, ON, Canada; 6 Department of Biomedical and Molecular Sciences, Queen’s University, Kingston, ON, Canada

**Keywords:** Stroke, Rehabilitation, Position sense, Proprioception, Robotics, Visuomotor

## Abstract

**Background:**

Several studies have found correlations between proprioception and visuomotor function during stroke recovery, however two more recent studies have found no correlation. Unfortunately, most of the studies to date have been conducted with clinical assessments of sensation that are observer-based and have poor reliability. We have recently developed new tests to assess position sense and motor function using robotic technology. The present study was conducted to reassess the relationship between position sense and upper limb movement following stroke.

**Methods:**

We assessed position sense and motor performance of 100 inpatient stroke rehabilitation subjects and 231 non-disabled controls. All subjects completed quantitative assessments of position sense (arm-position matching task) and motor performance (visually-guided reaching task) using the KINARM robotic device. Subjects also completed clinical assessments including handedness, vision, Purdue Pegboard, Chedoke-McMaster Stroke Assessment-Impairment Inventory and Functional Independence Measure (FIM). Neuroimaging documented lesion localization. Fisher’s exact probability tests were used to determine the relationship between performances on the arm-position matching and visually-guided reaching task. Pearson’s correlations were conducted to determine the relationship between robotically measured parameters and clinical assessments.

**Results:**

Performance by individual subjects on the matching and reaching tasks was statistically independent (Fisher’s test, P<0.01). However, performance on the matching and reaching tasks both exhibited relationships with abilities in daily activities as measured by the FIM. Performance on the reaching task also displayed strong relationships with other clinical measures of motor impairment.

**Conclusions:**

Our data support the concept that position sense deficits are functionally relevant and point to the importance of assessing proprioceptive and motor impairments independently when planning treatment strategies.

## Background

Sensory deficits are particularly common following stroke, occurring in up to 70% of patients [1]. Many studies have indicated a connection between impaired sensation and functional recovery of the upper extremity. In particular, proprioceptive deficits have been shown to negatively impact safety, postural stability and motor function [2]. Impaired proprioception has also been shown to have prognostic significance in self-care, likelihood of discharge home and length of hospital stay [3-7].

Although some reports have indicated that position sense, a sub-component of proprioception [8], strongly correlates with motor recovery of the hemiplegic arm after stroke [9-13], two studies have failed to support this relationship [14,15]. One of the studies [14] that failed to demonstrate a relationship used the Thumb Localizer Test [16] of proprioception while the other relied on a simple clinician administered two point rating scale (impaired vs. normal) [15]. These and similar clinical assessments of proprioception have been shown to have poor inter- and intra-rater reliability and tend to use coarse ordinal scales [17,18]. Recent technological advances have the potential to create better measurements of proprioception and motor function in patients with stroke [19-23]. We have developed reliable robotic assessments of position sense [24] and visuomotor function [25] in patients with stroke. This has created the opportunity to re-evaluate the relationship between proprioceptive, motor and functional deficits.

A better understanding of the relationship between proprioception and motor deficits is clinically important because it can guide rehabilitation. Traditionally, stroke rehabilitation has placed a significant focus on restoring motor function with little emphasis on recovery of proprioception. Clinically, we have observed patients with impaired proprioception who have sub-optimal functional recovery despite treatment with existing evidence-based motor rehabilitation treatments. A recent review of rehabilitation for sensory loss following stroke [26] examined a number of small studies, some of which demonstrate potential for clinical use. Many of these studies used motor outcome measures to evaluate passive sensory retraining treatments [27-34]. Given sensory and motor recovery may not be as strongly correlated as once thought, this strategy may be inappropriate.

A fundamental part of developing a treatment for stroke is to first evaluate the extent and nature of the stroke-related deficits. It is thus desirable to have appropriate tools for assessment of sensory impairments that are independent of motor assessment tools in order to interpret the effects of rehabilitation strategies on the sensory system. At the level of the individual patient, a more in-depth understanding of the patient’s exact impairments creates the ability to develop targeted and personalized rehabilitation strategies. For instance, two patients may present with the inability to perform a particular activity of daily living. Yet the reason for this may be related to impairment in sensation, motor function or both. Our anecdotal experience suggests that both these patients would receive the same “one size fits most” treatment approach in our centres. Better assessment may allow us to target treatment at one system versus the other, or both if necessary. The primary objective of the present study was to examine the relationship between upper extremity position sense deficits and visuomotor deficits following stroke. We used a robotic assessment of position sense and made comparisons to motor performance on a robotic visually-guided reaching task. Our secondary objective was to make comparisons of performance on the robotically administered assessments to more traditional clinical outcome tools: the Purdue Pegboard (PPB), the Chedoke-McMaster Stroke Assessment - Impairment Inventory (CMSA) of the arm and hand, and the FIM. Given that many groups have reported links between proprioception and motor outcomes, one might expect that subjects with stroke with impaired position sense should demonstrate impairments on many of these measures.

## Methods

### Subjects

Subjects with stroke were recruited from the inpatient stroke rehabilitation ward at Providence Care, St. Mary’s of the Lake Hospital site in Kingston, Ontario, Canada and the Foothills Medical Centre in Calgary, Alberta, Canada. Non-disabled control subjects were recruited from the communities of Calgary, Alberta, Canada or Kingston, Ontario, Canada. The ethics review boards of Queen’s University, Providence Care, and the University of Calgary approved the study. All subjects provided informed consent prior to participating in the study.

Subjects were included in the study if they were 18 years of age or older and were able to understand the instructions required to complete the assessments. Subjects were excluded from the study if they had ongoing musculoskeletal problems of the upper extremity or history of neurological disorders other than stroke. Subjects with stroke were also excluded if hemispatial neglect was confirmed by the conventional subset of the Behavioral Inattention Test (BIT).

Stroke lesion locations were documented using neuroimaging (Computed Tomography or Magnetic Resonance Imaging). Within the rest of this text, rather than referencing subjects with stroke by the side of their lesion, we refer to the *clinically most affected side of their body*.

### Robotic assessment

#### Robotic device

The robotic assessment was performed using the KINARM exoskeleton robotic device (BKIN Technologies Ltd., Kingston, Ontario) [35]. Subjects sat in a modified wheelchair base with each arm supported in the horizontal plane by the robotic exoskeleton. The device permits arm movements in the horizontal plane, monitors shoulder and elbow motion and can apply mechanical loads at the shoulder and/or elbow. Subjects were allowed free head movement and viewed an augmented reality system that could display visual targets in the same plane of the arms. Details of the tasks and analyses used in this study have been previously described [24,25].

#### Assessment of position sense: Arm-position matching task

**Figure 1:**
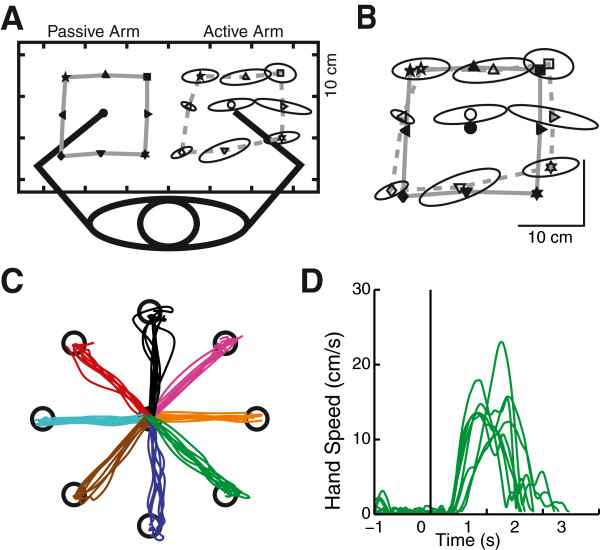
**Exemplar control subject data.A**) Arm-position matching task as seen from above. On the left, the positions of the robotically moved hand are filled symbols; positions of the actively moved hand are open symbols. Ellipses represent 1 standard deviation. **B**) Passive (robotically moved) hand positions have been mirrored onto those of the active hand for visualization purposes. **C**) Individual hand paths for movements to each of the eight targets in the visually guided reaching task. **D**) Velocity profiles for hand paths from the centre to the rightward target.

With the arms and hands occluded from vision, the robot passively moved one hand (passive arm) to one of nine spatial locations separated by 10 cm (Figure 1A,B). When the robot completed the passive movement, subjects actively moved their opposite hand (active arm) in an attempt to mirror match the passive hand position. Subjects were permitted as much time as necessary to match the passive hand location before cuing the experimenter to trigger the next trial. Each subject completed 6 randomized blocks for a total of 54 trials. For subjects with stroke, the robot passively moved the arm on their affected side. Controls were tested on both arms.

Three parameters quantified position sense: 1) trial-to-trial *Variability* of the active hand (Var_xy_), 2) *Contraction/Expansion* of the spatial area enclosed by the active hand relative to the spatial area enclosed by the passive hand (Area_xy_), and 3) *Systematic Shifts* between the passive and active hands (Shift_xy_). The formulae for these parameters have been previously described and provide reliable, 2-dimensional quantification of common deficits in position sense, and may describe different physiologic/functional attributes of limb position sense [24]. For this study, Contraction/Expansion was normalized by control performance to create positive scores for both contraction and expansion. Values between 0 and 1 indicated performance within the 95% confidence bounds of non-disabled controls, whereas values greater than 1 indicated abnormal contraction or expansion.

#### Assessment of motor performance: visually guided reaching task

With full vision of the arms and hands, subjects were instructed to reach “as quickly and accurately as possible” from a central target (near the centre of the arm’s workspace) to one of eight peripheral targets located 10 cm away (Figure 1C,D). The robot provided no active resistance or assistance for the reaching movements. Each trial began with the subjects holding their index finger tip at the central target for 1250–1750 ms before the peripheral target was illuminated thereby signaling to initiate their reaching movement. Subjects were provided 3000 ms to complete each reach. Each subject completed 8 randomized blocks. Two catch trials, in which no peripheral target was displayed, were included in each block. In total, each subject completed 80 trials with each arm.

We report five movement parameters, one from each of five attributes of sensorimotor control: a) postural control, b) reaction time, c) initial movement d) corrective movements, and e) total movement metrics. The algorithms for determining all parameters have been previously described in detail [25]. Postural control was characterized by postural hand speed (PS) for the 500ms prior to peripheral target illumination. Reaction time (RT) was the time from peripheral target onset until movement onset. The initial phases of movement from movement onset to the first speed minimum were quantified using initial movement direction error (IDE). IDE is the angular deviation between a straight line from the central to peripheral target and the actual path taken in the initial phase of movement (first minima of hand speed after movement onset). Corrective movements after their initial response were quantified by recording the number of speed peaks per movement (NSP). The total movement metric used was total movement time (MT). These parameters provided the best combination of high reliability and sensitivity (ie. the ability to detect the presence of an impairment relative to normal behaviour) for each attribute [25].

#### Clinical assessment

The clinical assessment consisted of a broad range of sensory, motor, and functional evaluations. These measures were chosen as the majority of them are used in day-to-day practice in our stroke rehabilitation centres.

Handedness was documented with the Modified Edinburgh Handedness inventory [36]. Subjects were considered Right handed (R) if they scored >50, Left handed (L) if they scored <−50 and Mixed handedness (M) if they scored >−50 and <50.

Position sense was assessed clinically with the Thumb Localizer Test (TLT) [16], chosen because of its previous use quantifying whole-limb position sense in several studies of subjects with stroke [14,37-43]. As described by Hirayama [16], the subject, with eyes closed, extends one thumb and the examiner moves that arm to a position in front of the subject at or above eye level, lateral to the midline. The subject is then asked to pinch the extended thumb with the opposite thumb and forefinger. Subjects are scored 0 (accurately does the task) to 3 (subject is unable to find his or her thumb and does not climb up the affected arm to locate it). Subjects with stroke localized the thumb on their affected arm whereas controls localized the thumb on their dominant arm.

Spasticity was assessed using the Modified Ashworth Scale [44]. In this assessment, the examiner moves the subject’s limb through a range of motion and evaluates the resistance to passive movement. Scores range from 0 (no increase in muscle tone) to 4 (affected part is rigid in flexion or extension). In the present manuscript we report the MAS for the elbow flexors.

Motor impairment was assessed using the Purdue Pegboard (PPB) (LaFayette Instrument Co., LaFayette, IN, USA) [45]. In this assessment the subject attempts to place as many pegs as possible in a board in a 30 second time window with one hand. While this is traditionally touted as a test of fine motor skills, the subject is required to use the proximal upper extremity to get the hand to the correct position to retrieve and insert each peg.

The Chedoke-McMaster Stroke Assessment - Impairment Inventory (CMSA) [46] was performed to stage the arm and hand. This tool relies on the concept of stages of motor recovery, first introduced by Twitchell [1], whereby a patient is thought to progress through a sequence of stages from flaccid paralysis (Stage 1) to normal movement (Stage 7) as they recover from a stroke. Individuals are scored based on their ability to perform a set of predetermined movements in front of an examiner. The authors of the tool indicate that the “Stages of Motor Recovery” serve as a measurement for the amount of neurological impairment.

Functional abilities were documented with the Functional Independence Measure (FIM) [47]. This 18 item scale scores individuals across a number of items. There are 13 items considered to be motor tasks (eating, grooming, bathing, upper and lower body dressing, toileting, bladder and bowel management, bed to chair transfers, toilet and shower transfers, locomotion and the ability to do stairs). There are 5 items considered to be cognitive tasks (cognitive comprehension, expression, social interaction, problem solving and memory). Each item is ranked on a 7-point ordinal scale that ranges from 1 (total dependence) to 7 (complete independence). In the present manuscript we present the total FIM score (measured out of 126) and the FIM self-care subscore (FIMsc – measured out of 42, items include: eating, grooming, bathing, dressing above the waist, dressing below the waist and toileting).

Visual acuity was assessed with a Snellen eye chart and visual fields were examined using confrontation [48]. Visuospatial attention was assessed using the conventional subset of the Behavioural Inattention Task (BIT) [49]. The BIT is a standardized assessment for visuospatial inattention (also known as hemispatial neglect) with a number of pencil and paper tests including line, letter and star cancellation, line bisection, figure copying and drawing.

Stroke lesion locations were recorded by one of the study physicians with experience in neuroimaging (S.P.D). Clinical computed tomography (CT) or magnetic resonance imaging (MRI) scans and the clinical reports generated by a neuroradiologist were evaluated. From this a standard form was used to document the side of the lesion, the vascular territory (eg. right middle cerebral artery), and brain region (egs. cortical, subcortical, brainstem, cerebellum).

Non-disabled controls completed the same assessments as subjects with stroke with a few exceptions. No control subject performed the FIM or BIT. A subset of non-disabled controls performed the TLT, MAS and CMSA to obtain some idea about the amount of variability amongst controls in these clinical measures.

A trained study physician or a physiotherapist with expertise in stroke rehabilitation performed all clinical assessments.

#### Statistical comparisons

Statistical Analyses were performed using MATLAB (Mathworks, Inc., Massachusetts, USA). Subjects with stroke were flagged for impairments on robotic parameters when their performance fell outside a parameter’s normative reference range, defined as the 95% confidence boundaries of control performance taking age [50-53], sex [54-56], and test-arm (dominant/non-dominant) [57-62] into consideration. Our primary analysis was concerned with the performance of the most affected arm in subjects with stroke. To establish each parameter’s normative reference range, control values were tested for normality (Lillifors test, *P*< 0.01) and transformed with a log, square root, or inverse transform to obtain normality when necessary. Regression analyses were then used to model the effects of age, gender and test-arm when creating normative reference ranges for each stroke subject.

Impairments on the parameters from each task were used to classify whether a subject had performed “normally” or “abnormally” on either the arm position matching task, the visually-guided reaching task, both or neither. Fisher’s exact probability tests and Pearson’s correlation tests were used to quantify relationships between robotic and clinical measures. The *p* value of significance on these tests was set at *p* < 0.01. Bonferroni corrections were applied to tests with multiple comparisons.

## Results

### Subject pool

Data were collected from 100 subjects with stroke and 231 non-disabled controls. Demographic and clinical features of the groups are provided in Table 1. Left- and right-affected stroke groups were similar with respect to age, gender, handedness and time since stroke. Spasticity of the affected arm, as clinically measured by the MAS, was evident in about half the subjects with stroke (n = 48), but was typically mild or moderate in severity (1, 1+, or 2, n = 46). FIM scores were similar between left- and right-affected stroke groups (Wilcoxon rank sum test, *P* = 0.07). While most FIM scores fell within the mild range for stroke severity (FIM >= 80, n = 78), many were found within the moderate and severe ranges (FIM < 80, n = 22). Eleven left- and eight right-affected subjects with stroke demonstrated evidence of hemianopsia during confrontation testing. No subjects with evidence of hemispatial neglect (BIT < 130) were included in the present study.

**Table 1:** Subject characteristics

**Measure**	**Group**		
	**Stroke, Left Affected {n = 46}**	**Stroke, Right Affected {n = 54}**	**Control {n = 231}**
Age	64 (22 – 90)	62 (21 – 84)	48 (20 – 88)
Gender	25 M, 21 F	32 M, 22 F	108 M, 123 F
Handedness	41 R, 1 L, 4 M	41 R, 7 L, 6 M	208 R, 14 L, 9 A
Type of stroke	41 ischemic, 5 hemorrhagic	42 ischemic, 12 hemorrhagic	—
Days since stroke	25 (5 – 75)	31 (6 – 81)	—
Thumb Localizing Test [0–3]	[19,11,10,3] {n = 43}	[30,14,5,1] {n = 50}	[92, 9, 6, 0] {n = 107}
CMSA affected arm [1-7]	[3,5,5,5,7,7,14]	[2,5,9,2,13,8,15]	—
CMSA unaffected arm [1-7]	[0, 0, 0, 0, 1, 8, 37]	[0, 0, 0, 0, 1, 12, 41]	[0, 0, 0, 0, 0, 0, 108] {n=108}
MAS affected arm [0–4]	[26, 10, 5, 4, 1, 0]	[24, 19, 6, 2, 1, 0] {n=52}	—
MAS unaffected arm [0–4]	[42, 4, 0, 0, 0, 0]	[51, 1, 0, 0, 0, 0] {n=52}	[98, 0, 0, 0, 0, 0] {n = 98}
PPB affected arm	3 (0 – 11)	4 (0 – 15)	—
PPB unaffected arm	11 (4 – 16)	11 (5 – 19)	14 (8 – 20) {n = 205}
FIM [18–126]	93 (53 – 124)	104 (43 – 126)	—
FIMsc [6-42]	30 (12 – 42) {n=45}	35 (13 – 42)	—
BIT [0–146]	143 (130 – 146)	143 (130 – 146)	146 (137 – 146)
Visual Field Defects	11 Y, 35 N	8 Y, 46 N	

A small number of clinical assessments were not carried out on all subjects. For those assessments with missing subjects, the actual number of subjects is indicated within curly brackets, {}. Variables including age, days since stroke, Purdue Pegboard (PPB), Functional Independence Measure (FIM), FIM self care subscore (FIMsc), and Behavioral Inattention Test (BIT) are indicated as the median followed by the range within round brackets, (). Variables including Thumb Localizing Test, Chedoke-McMaster Stroke Assessment - Impairment Inventory (CMSA), and Modified Ashworth Scores (MAS) are shown as the number of subjects within each category within square brackets, []. For example, there are 6 values for the MAS, corresponding to the number of subjects that scored a 0, 1, 1+, 2, 3, or 4. Thumb localizing scores indicate the score when subjects were localizing their affected/dominant thumb with their unaffected/non-dominant hand. CMSS, MAS and PPB scores of controls are for the dominant arm. Abbreviations: M – Male, F – Female, R – Right handed, L – Left handed, Mi – Mixed handedness.

### Robotic data

#### Individual subject exemplars

Figure 1A,B shows arm position matching results of a healthy 67 year old female control subject. The robot passively moved the left arm and the subject actively moved the right arm to the mirror location in space. As with most non-disabled controls, variability about each target is relatively small (< 5 cm) and there is little spatial contraction/expansion or systematic shift. Figure 1C,D depicts reaching performance of the same subject. The modest curvatures in the hand-paths (Figure 1C) and moderate variability in the velocity profiles (Figure 1D) are typical for a non-disabled control subject of this age.

Table 2 provides the actual values for the robotic parameters for the 67 year-old right handed female depicted in Figure 1. The robotic parameters that demonstrated a significant effect of age, sex or test-arm in the non-disabled controls are also identified with an X in Table 2. Further, the median, interquartile range and normative reference range (parameter values at the limit of what is considered to be within the control range) are presented for both a 27 year-old and a 67 year-old right-handed female for comparison purposes. Notice the specific differences in the normative reference ranges between the two.

**Table 2:** Significant effects of age, sex, and test-arm for each parameter with the corresponding normative statistics at two distinct ages, 27 and 67 years old

**Parameter**	**67 Year Female Exemplar Subject (Left, Right)**	**Significant Effects**			**Normative Statistics (Age = 67)**			**Normative Statistics (Age = 27)**		
		**Age**	**Sex**	**Arm**	**Median**	**IQR**	**NRR**	**Median**	**IQR**	**NRR**
PS (cm/s)	0.324, 0.352	X	—	—	0.425	0.219	< 0.752	0.291	0.151	< 0.516
RT (s)	0.386, 0.380	X	—	—	0.373	0.069	< 0.484	0.319	0.050	< 0.397
*MT (s)	1.182, 1.286	X	X	—	1.136	0.203	< 1.425	1.071	0.203	< 1.360
IDE (deg)	3.003, 2.225	X	—	—	2.660	0.897	< 4.141	2.185	0.737	< 3.403
*NSP	1.952, 2.516	—	X	—	2.195	0.492	< 2.845	2.195	0.492	< 2.845
†Var (cm)	4.868, 4.466	X	—	X	3.824	1.127	< 5.695	3.225	0.951	< 4.802
C/E	1.015, 0.968	X	—	—	0.766	0.292	0.415 - 1.199	0.875	0.292	0.523 - 1.307
Shift (cm)	1.550, 1.585	X	—	—	4.106	3.264	< 8.776	3.534	3.030	< 7.929

* Normative statistics are shown for female participants only.

† Normative statistics are shown for the dominant arm only.

Abbreviations: IQR – Interquartile range, NRR – Normative Reference Range.

**Figure 2:**
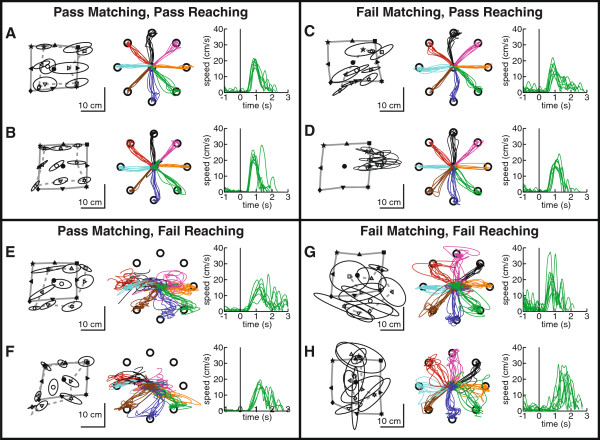
**Exemplar data from subjects with stroke.** Data are presented in the left column for position matching, and the middle and right hand columns for visually guided reaching movements. **A** &**B**) Two subjects with stroke who performed within normal limits on both tasks. **C** &**D**) Two subjects with stroke who performed outside the normal range on the matching task, but were normal on the reaching task. **E** &**F**) Two subjects with stroke who performed normally on the matching task, but were abnormal on the reaching task. **G** &**H**) Two subjects with stroke who performed abnormally on both tasks.

Figure 2 illustrates the performance of subjects with stroke in the matching and reaching tasks. These exemplar subjects illustrate that statistically established deficits in the matching task are not necessarily coupled with significant deficits in the reaching task, and vice versa.

Figure 2A,B displays two exemplar subjects with stroke whose performance was not statistically different from non-disabled controls. Figure 2A shows a right-affected subject with an ischemic stroke of the left pontine artery 28 days prior to robotic testing. A right-affected subject who experienced a hemorrhagic stroke of the left basilar artery 29 days before robotic assessment is illustrated in Figure 2B. Despite their different stroke types, their reaching and matching performance is qualitatively and quantitatively similar to controls.

Other subjects with stroke performed abnormally on the matching task but normally on the reaching task (Figure 2C,D). Figure 2C depicts a right-affected subject with an ischemic stroke of the left anterior cerebral artery 9 days prior to robotic testing. This subject demonstrates a significant systematic shift in the matching task, yet is unimpaired on the reaching task. Figure 2D depicts data from a left-affected subject who had an ischemic stroke of the right middle cerebral artery (MCA) 59 days before robotic examination. This subject exhibits significantly increased variability in the matching task, but normal reaching behaviour with the affected left arm.

Another group of subjects with stroke performed normally on the matching task, but abnormally on the reaching task (Figure 2E,F). Figure 2E displays a left-affected subject with an ischemic stroke of the left pontine artery 24 days before robotic testing. Figure 2F illustrates a left-affected subject with an ischemic stroke of the right MCA 24 days prior to robotic assessment. These subjects perform qualitatively and quantitatively similar to controls in the matching task. However, there are clear abnormalities in the hand paths (middle) and multiple peaks in the velocity profile (right) consistent with deficits in reaching.

A final group of subjects with stroke performed abnormally on both robotic tasks. Figure 2G depicts this pattern in a subject with an ischemic left MCA stroke 15 days prior to robotic examination. Figure 2H shows a subject with an ischemic right PCA stroke 19 days prior to robotic assessment.

#### Stroke group data

Our key observation around our primary objective was that deficits in arm-position matching were largely independent of deficits in visually-guided reaching across the group studied. Table 3 displays the number of subjects found in each category (deficits in matching, reaching, neither or both) and these numbers are not significantly different from those predicted if the two behaviours were completely independent of each other (Fisher’s exact probability test, *P* = 0.12).

**Table 3:** Relationship between the matching and reaching tasks and clinical measures

**Group**	*Subjects	**TLT**	**PPB**	**CMSA Arm**	**FIM**	**FIMsc**
Neither	5	[4,0,0,0]	8 (3-15)	[0,0,0,1,1,0,3]	119 (106-122)	42 (35-42)
Match Only	5	[2,0,2,1]	10 (9-11)	[0,0,0,0,1,1,3]	93 (82-112)	30 (27-38)
Reach Only	33	[23,6,3,0]	4 (0-13)	[2,3,3,1,6,6,12]	103 (74-126)	37 (16-42)
Both	57	[20,19,10,3]	2 (0-11)	[3,7,11,5,12,8,11]	99 (43-124)	31 (12-42)

* Reaching and matching deficits exhibit independence (Fisher’s Test, *P* = 0.12).

**Figure 3:**
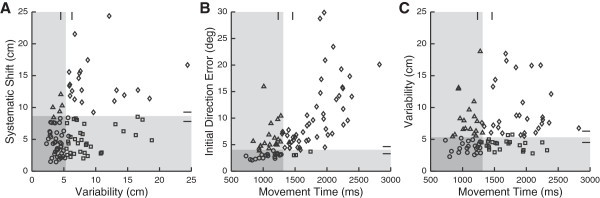
**A) Scatter plot of matching parameters Variability (Var**_**xy**_**) vs Systematic Shift (Shift**_**xy**_**).** Grey shading represents normal control performance on each parameter. Tick marks indicate regions where normal performance can vary due to each subject’s age, gender, and/or active arm. Circles: subjects with stroke who perform normally on both parameters. Diamonds: subjects with stroke who perform abnormally on both parameters. Squares: subjects with stroke who perform abnormally on Var_xy_ only. Triangles: subjects with stroke who perform abnormally on Shift_xy_ only. **B**) Scatter plot of reaching parameters Movement Time (MT) versus Initial Direction Error (IDE). Shading and symbols similar to above. **C**) Scatter plot of reaching parameter MT versus position matching parameter Var_xy_. Shading and symbols similar to above.

A more detailed demonstration of this independence is seen by examining the categorical relationships (Fisher’s exact probability tests) and correlation coefficients (Pearson’s correlations) of individual parameters within and between the tasks (Table 4). In the matching task, Fisher’s tests demonstrated significant categorical relationships between Variability, Contraction/Expansion, and Systematic Shifts (All *P* < 0.0014). Additionally, Pearson’s correlations revealed significant relationships between Variability and Shift (*P* < 0.0014) with a correlation of 0.36. Figure 3A illustrates the relationship between Variability and Systematic Shifts. Far more subjects failed (diamonds) or passed (circles) on both parameters than expected by random chance, whereas far fewer subjects had deficits in only one of the two parameters (squares, triangles).

**Table 4:** Relationships between matching and reaching measures

	**Var**_**xy**_	**Area**_**xy**_	**Shift**_**xy**_	**PS**	**RT**	**IDE**	**NSP**	**MT**
**Var**_**xy**_		0.18	**†0.36**	0.01	0.29	0.13	0.21	0.19
**Area**_**xy**_	**†<10**^**-5**^		0.13	0.23	-0.02	0.12	0.09	0.01
**Shift**_**xy**_	**†<10**^**-3**^	**†<10**^**-3**^		0.05	0.14	0.28	0.27	0.28
**PS**	0.301	0.024	**†<10**^**-3**^		–0.04	0.12	0.03	–0.23
**RT**	0.004	0.128	0.003	0.014		**†0.36**	0.28	**†0.36**
**IDE**	0.031	0.326	0.024	0.069	**†<10**^**-3**^		**†0.62**	**†0.67**
**NSP**	0.006	0.080	0.087	0.378	0.027	**†<10**^**-5**^		**†0.78**
**MT**	0.047	0.341	0.033	0.119	**†<10**^**-3**^	**†<10**^**-8**^	**†<10**^**-8**^	

Lower left: Probabilities of independence between categorical contingencies (Fisher’s exact probability tests). Upper right: Correlations between measures (Pearson coefficients). †Significant contingencies and correlations, *P* < 0.0014 (Bonferroni’s method is used to control for multiple comparisons; α=.01, n = 7).

Within the reaching task, a similar trend was observed across parameters except for relationships with PS (Table 4). The relationship between the parameters MT and IDE is shown in Figure 3B. Similar to the matching task, more subjects exhibited deficits (diamonds) or were normal (circles) on both parameters and fewer subjects showed deficits in only one of the two parameters (squares, triangles) than would be expected by chance alone (Fisher’s test, *P* < 0.0014). Additionally, there was a significant Pearson’s correlation between these two parameters (*r* = 0.67, *P* < 0.0014). Except for the weak relationship between RT and NSP, all other relationships between RT, MT, IDE, and NSP showed statistical categorical results (Fisher’s tests, *P* < 0.0014).

In contrast to the relationships within each task, there was substantively less statistical interactions between the matching and reaching tasks (Table 4). The relationship between Variability (matching) and Movement Time (reaching) is illustrated in Figure 3C. The number of subjects identified as performing abnormally on both parameters, one only or neither was essentially randomly distributed (Fisher’s test, *P* = 0.047) and the Pearson’s correlation was not significant (*r* = 0.19, *P* > 0.0014). In fact, only one pair of reaching and matching parameters, Shift_xy_ and PS, exhibited a significant categorical relationship (Fisher’s test, *P* < 0.0014) and none of the matching and reaching parameters demonstrated a significant Pearson’s correlation (all *P* > 0.0014).

#### Effect of visuomotor impairment of the ipsilesional arm

A small number of subjects with stroke in the present study (n = 9) demonstrated impairment in the ipsilesional (less affected) arm that caused them to have difficulty getting to visually guided reaching targets in more than 5% of trials. As part of a secondary analysis, we removed these individuals from the data set and again compared the relationship between the visually guided reaching task and the position matching task. We observed that deficits in arm position matching were still largely independent of those in visually guided reaching (Fisher’s test, *P* = 0.22).

#### Relationship between robotic assessment and clinical measures of impairment and disability

Although there were minimal interactions between the matching and reaching tasks, both tasks often correlated with clinical measures of impairment and disability (Table 5). All three matching parameters had a significant categorical relationship with FIM (Fisher’s test, *P* < 0.0013) while Variability and Systematic Shifts also exhibited significant Pearson’s correlations (*P* < 0.0013). Except for Reaction Time, the reaching parameters also showed either a significant categorical relationship (PS, IDE: Fisher’s test, *P* < 0.0013) and/or Pearson’s correlation (IDE, NSP, MT: *P* < 0.0013) with the FIM. Similarly, some reaching parameters also displayed significant categorical relationships with the Purdue Pegboard (RT: Fisher’s test, *P* < 0.0013) and Chedoke-McMaster Stroke Assessment - Impairment Inventory (RT, MT: Fisher’s tests, *P* < 0.0013). Further, most reaching parameters displayed Pearson’s correlations (RT, IDE, NSP, MT: *P* < 0.0013) with the Purdue Pegboard. The Thumb Localizing Test had significant categorical relationships with Var_xy_ and Shift_xy_ (Fisher’s test, *P* < 0.0013). However, it also showed a significant categorical relationship with NSP (Fisher’s test, *P* < 0.0013).

**Table 5:** Relationships between individual robotic measures and clinical measures

**Clinical Measure**	**Category (Fisher’s Test)**		**Parameter**							
	**Normal**	**Impaired**	**Var**_**xy**_	**Area**_**xy**_	**Shift**_**xy**_	**PS**	**RT**	**IDE**	**NSP**	**MT**
**FIM**	**x ≥ 80**	**x < 80**	**†<10**^**-3**^	**†<10**^**-3**^	**†<10**^**-3**^	**†<10**^**-3**^	0.028	**†0.001**	0.015	0.049
**PPB**	**x ≥ 10**	**x < 10**	0.067	0.307	0.095	0.144	0.002	0.010	0.024	**†<10**^**-3**^
**CMSS**	**x = 7**	**x < 7**	0.081	0.178	0.017	0.093	**†<10**^**-3**^	0.007	0.014	**†<10**^**-3**^
**TLT**	**x = 0**	**x > 0**	**†<10**^**-4**^	0.285	**†<10**^**-3**^	0.153	0.042	0.027	**†<10**^**-3**^	0.005
**FIM**			**†–0.32**	–0.12	**†–0.41**	–0.17	–0.21	**†–0.40**	**†–0.38**	**†–0.41**
**FIMsc**			–0.22	–0.05	**†–0.38**	–0.19	–0.20	**†–0.43**	**†–0.42**	**†–0.43**
**PPB**			–0.22	–0.04	**†–0.33**	–0.15	**†–0.35**	**†–0.57**	**†–0.48**	**†–0.61**

Top: probabilities of independence between categorical contingencies of individual robotic measures and clinical assessments (Fisher’s exact probability test). Bottom: Pearson correlations between robotic measures and clinical assessments. Fisher’s tests include all subjects whereas correlation coefficients exclude subjects that were unable to do the reaching task. †Significant contingencies and correlations, *P* < 0.0013 (Bonferroni’s method is used to control for multiple comparisons; α = 0.01, n = 8).

#### Relationship between lesion location and performance on robotic measures

Taking into account the side, vascular territory and the cortical or sub-cortical nature of the stroke lesions, we assessed the lesions to determine whether certain locations might be more commonly associated with deficits in the two tasks. A large cohort of our subjects with stroke had cortical or sub-cortical lesions of the MCA territory (29 right and 31 left). Subjects with identified deficits for reach only, match only and both tasks were 4, 3 and 21 for right MCA lesions and 13, 0 and 17 for left MCA respectively. More subjects displayed deficits in reach only with left MCA lesions compared to right MCA lesions, the reverse was true for match only deficits. However, these differences did not reach statistical difference (Fisher’s test, p = 0.98). As well, the distribution of deficits across the two tasks for subjects with lesions in the MCA territory were also not different than the distributions observed for the remaining subjects with lesions elsewhere in the brain (Fisher’s test, p = 0.11).

## Discussion

Our findings point to the fact that position sense, as assessed by the arm-position matching task, was largely independent of deficits in motor performance, as assessed by the visually-guided reaching task. Importantly, deficits in position sense were significantly correlated with the FIM. The FIM is a rehabilitation instrument that measures functional abilities [47] and is widely used by stroke rehabilitation clinicians in North America. Thus, we included it in the present study. However, the FIM is, perhaps, not the most sensitive instrument for upper-limb function, as many domains do not include upper limb function. Further, the FIM collapses across both motor and cognitive function.

Despite the above caveats, our findings still imply that impaired position sense has an impact on daily activities, something that mirrors our clinical experience. This point is important because many traditional rehabilitation strategies [63] focus heavily on remediating or compensating for motor deficits. In contrast, sensory deficits typically receive little attention and yet a substantial proportion of subjects display impairments in proprioception. This raises the following question: if proprioceptive deficits are so common and interfere with function, why do clinicians often choose to ignore them in rehabilitation? Some may put forward that a substantial amount of evidence exists for rehabilitating motor function after stroke while there is little evidence for techniques aimed at improving sensory function. Perhaps the problem and the circularity of this argument come back to assessment. It is difficult, if not impossible, to demonstrate efficacy in a clinical study without an appropriate assessment tool. Tests of proprioceptive function have typically been unreliable [17]. In clinical practice, we have seen patients with large deficits in position sense and clinicians who incorrectly attributed the patient’s difficulty to issues with muscle tone or visual spatial processing. The present results suggest that rehabilitation therapy should address proprioceptive dysfunction and also explore new treatments for these deficits [18,22-24].

In addition to the FIM, we also found significant relationships between reaching performance and other clinical measures of movement. Our findings parallel those by Bosecker et al. [21] and add to the validity of using robotics for post-stroke assessment. The robotic technique has many strengths including objectivity, reliability and the fact it does not rely on an ordinal scale. The present study demonstrated greater sensitivity of the robotic technique for detecting motor impairments than a more traditional clinical assessment, the CMSA. This is highlighted by the 23 subjects with stroke who had normal CMSA scores but detectable abnormalities in the robotic reaching task.

More generally, robotic assessment may have many benefits and potential uses [19]. Monitoring the effectiveness of an intervention could be relatively easy, especially in situations where typical clinical scales suffer from poor sensitivity to change and reliability. Robotic assessments may provide insight into potential areas for targeted therapeutics, as the assessments can be designed to more specifically and precisely interrogate deficits than is possible with the naked eye. It is not uncommon in our clinical practice to encounter individuals after stroke that complain of subtle impairments that are not well elucidated by existing clinical measures. Robotic assessments may be helpful in objective identification of these impairments and aid in justifying further treatment. Importantly, for studying interventions, robotic testing has the potential to rapidly and reliably identify individuals with and without certain deficits, thus allowing the possibility of selection of a more homogenous study population.

We included the Thumb Localizer Test in the present study as it was the closest clinical standard for assessing whole upper extremity position sense reported in the literature. Our robotic measure of position sense exhibited a significant relationship with the TLT [16], but the two tests did not always identify abnormalities in the same subjects. Admittedly, there are differences in the conduct of the two tests. Unlike the arm-position matching task, subjects must cross the midline in the TLT. Some caution must be taken in interpreting the present results as versions of the TLT with a binary outcome scale have been shown to have poor reliability [17].

The data in this paper are from a heterogeneous group of sub-acute stroke rehabilitation patients. Inclusion criteria were specifically selected so that study subjects would appropriately represent individuals from sub-acute in-patient stroke rehabilitation. These criteria were chosen to aid in the generalizability of the results. We acknowledge our analysis of the imaging data was relatively simplistic at the present time, focusing only on the vascular territory involved in the stroke. Future studies with more detailed anatomic and volumetric analyses of lesions may be helpful in shedding light on the neuroanatomic correlates of impairments in position sense and visually guided reaching, but are beyond the scope of the present paper.

In the present study we collapsed our measures across target locations in both the position matching task and visually guided reaching tasks. A few recent reports in healthy individuals have indicated that targets located closer to the body are more clearly represented in the brain [64,65]. Determining what impact this may or may not have on the relationship between the two tasks was also considered beyond the scope of the present manuscript, but does highlight the potential for future study.

The robotic device used in the present study does not allow for sensorimotor assessment of the hand. However, we showed significant relationships between the robotic measures and the FIM as well as the FIM self-care subscore implying the potential importance of proximal limb impairments in overall function. This is consistent with a recent study demonstrating that the initial level of impairment in the proximal upper extremity is predictive of overall upper limb recovery after stroke [66].

The present robotic device functions in the horizontal plane. This position has been recommended for performing assessments in the upper limb as the device supports the limb against gravity [67]. Thus, patients with weakness following stroke are able to engage in assessments that would not be possible otherwise. This is highly advantageous in the clinical setting where early assessment may hold the key to important prognostic information. While three-dimensional assessment of position sense with gravitational support may be possible in more costly and complicated devices, it is not clear that it would add substantively to our ability to detect position sense impairments. This is because the primary peripheral detector for position sense is thought to be the muscle spindle [68,69] and most muscles are stretched in both horizontal and vertical movements. This fact, and our clinical experience with the current robotic technique lead us to believe the present results are generalizable to movements in three dimensions.

Due to the very nature of the position sense task, the robot passively moved the subject’s relaxed arm, thus depriving subjects of efference copy signals that are present in neurologically intact individuals during active movement. One could argue that position sense in subjects with stroke should improve if they have access to this information. However we did not specifically evaluate this and would require a different experimental paradigm aimed at determining the relative contributions of afferent information and efference copy.

As the present study featured prospective data collected at a single time point following stroke, it is difficult to predict whether the relationships we observed will be preserved over the entire trajectory of stroke recovery. Investigations with a longitudinal study would be able to quantify the nature of the relationship of proprioception and motor function over time and improve our understanding of the trajectory of proprioceptive recovery following stroke.

Despite the limitations listed above, the separation of deficits of position sense and those in reaching movements is somewhat surprising given the importance of proprioceptive feedback in voluntary motor control [70]. At one level, the independence of deficits on the matching and reaching task might have resulted from the fact that the matching task is a bimanual activity that requires more interhemispheric transfer than the unimanual reaching task. Further, the matching task requires an additional spatial transformation to “flip” the coordinates of the target to the opposite side of the workspace for matching. Thus, the separation between the two tasks may be a reflection of the more complex spatial demands of the matching task versus the reaching task.

However, another possibility is the suggestion of a potential distinction between using limb afferents for perception (position sense) versus using them for action (feedback control), akin to the distinction between the use of vision for perception and action [71]. Goodale and Milner [71] proposed two different streams for processing visual information, one stream responsible for processing information for the purpose of perception and a second stream responsible for processing visual information needed to undertake action. Studies in vision have largely supported this conceptual framework [72,73]. Although our data is suggestive of a similar separation in the use of limb afferents for perception versus for action, the reaching task involved the use of vision that may have compensated for impairments in the use of limb afferent information. Thus, further refinement of the motor task to remove the use of vision is necessary to verify if there is a distinction between perception and action for limb afferents.

## Conclusions

In the present study group, performance on the position sense task was independent of that on the visually guided reaching task. It also appears that position sense deficits impact performance of activities of daily living. This study points to the importance of assessing proprioceptive and motor impairments independently when planning treatment strategies or measuring the outcomes of interventions.

## Abbreviations

KINARM: Kinesiological Instrument for Normal and Altered Reaching Movements; Var_xy_: Variability; Area_xy_: Contraction/Expansion; Shift_xy_: Systematic Spatial Shift; PS: Postural hand Speed; RT: Reaction Time; IDE: Initial Direction Error; NSP: Number of Speed Peaks; MT: Movement Time; MAS: Modified Ashworth Scale; TLT: Thumb Localizer Test; PPB: Purdue Peg Board; CMSA: Chedoke-McMaster Stroke Assessment - Impairment Inventory; FIM: Functional Independence Measure; FIMsc: Functional Independence Measure self-care subscore; BIT: Behavioural Inattention Task; R-MCA: Right Middle Cerebral Artery; L-MCA: Left Middle Cerebral Artery; R-PCA: Right Posterior Cerebral Artery.

## Competing interests

SHS is the co-founder and scientific officer of BKIN technologies, the company which commercializes the KINARM robotic device. The other authors SPD, TMH and SDB have no competing interests to declare.

## Authors’ contributions

SPD participated in the design of the study, data collection, data analysis and drafting the manuscript. TMH carried out data analysis and assisted with drafting of the manuscript. SDB was involved with patient recruitment and drafting of the manuscript. SHS was involved with design and coordination of the study, data analysis and drafting of the manuscript. All authors read and approved the final manuscript.

## Authors’ information

SPD is a practicing Physiatrist who specializes in stroke rehabilitation. He routinely sees patients with sensory deficits following stroke.

## Funding

This work was supported by a CIHR operating grants (MOP 81366, MOP 106662, NSP 104015) and a Heart and Stroke Foundation of Alberta, NWT and Nunavut Grant-in-Aid. S.P.D. was supported by an Alberta Nunavut and N.W.T. Heart and Stroke Foundation Investigatorship in Stroke Rehabilitation Research.
